# Observation of exceptionally strong near-bottom flows over the Atlantis II Seamounts in the northwest Atlantic

**DOI:** 10.1038/s41598-024-60528-2

**Published:** 2024-05-05

**Authors:** Oleg A. Godin, Tsu Wei Tan, John E. Joseph, Matthew W. Walters

**Affiliations:** 1 Physics Department, Naval Postgraduate School, Monterey, CA 93943 USA; 2 Department of Marine Science, ROC Naval Academy, Kaohsiung, 81345 Taiwan; 3 Oceanography Department, Naval Postgraduate School, Monterey, CA 93943 USA

**Keywords:** Ocean sciences, Physics

## Abstract

Knowledge of near-bottom ocean current velocities and especially their extreme values is necessary to understand geomorphology of the seafloor and composition of benthic biological communities and quantify mechanical energy dissipation by bottom drag. Direct measurements of near-bottom currents in deep ocean remain scarce because of logistical challenges. Here, we report the results of flow velocity and pressure fluctuation measurements at three sites with depths of 2573–4443 m in the area where the Gulf Stream interacts with the New England Seamounts. Repeated episodes of unexpectedly strong near-bottom currents were observed, with the current speed at 4443 m of more than 0.40 m/s. At 2573 m, current speeds exceeded 0.20 m/s approximately 5% of the time throughout the entire eight-week measurement period. The maximum flow speeds of over 1.10 m/s recorded at this site significantly surpass the fastest previously reported directly measured current speeds at comparable or larger depths. A strong correlation is found between the noise intensity in the infrasonic band and the measured current speed. The noise intensity and the characteristic frequency increase with the increasing current speed. Machine-learning tools are employed to infer current speeds from flow-noise measurements at the site not equipped with a current meter.

## Introduction

Near-bottom currents play a key role in a number of fundamental physical and geological processes in the ocean, including sediment transport^1–3^, shaping the seafloor morphology and creation of specific bedforms^2,4,5^, development and dynamics of nepheloid layers^3^, and dissipation of kinetic energy by bottom drag^6^. In biological and ecological context, near-bottom currents are widely recognized as a major driver of the composition, diversity, and abundance of benthic fauna^7,8^.

For acoustic and seismic measurements with sensors at or near the seafloor, these flows often contribute significantly to the noise floor and may limit the sensor performance^9–12^. Swift near-bottom currents strongly tilt and can drag various moorings^1,11^. In industrial applications, estimates of extreme near-bottom currents and the stresses they create are required to design, assess survivability, and insure underwater installations such as pipelines and communication and power cables^13^.

Near-bottom currents with speeds exceeding several meters per second are known to occur close to shore especially at constrained passages such as Gibraltar Strait^14^. The measurements are more difficult, and the data are scarce in the deep ocean. To our knowledge, the swiftest near-bottom currents at depths over 2000 m were directly measured on the continental rise off Nova Scotia^1,15^ and in Drake Passage^16^. Richardson et al.^15^ reported maximum current speed of 0.73 m/s at depth of 5022 m. Very strong near-bottom currents at depths between 3000 and 4000 m were measured at several points in the Antarctic Circumpolar Current in Drake Passage, with the maximum reported speed of 0.78 m/s^16^.

This paper reports observations of episodes of exceptionally strong near-bottom currents (benthic storms^1,17^) at depths exceeding 2500 m. These extreme abyssal currents were encountered on the flanks of the Atlantis II Seamounts in proximity to the Gulf Stream. The peak current speeds exceed 1.1 m/s and are faster than any directly measured currents that were previously reported in the literature for such depths. We also report concurrent continuous acoustic measurements of underwater noise over a nearly two-month period. The acoustic measurements revealed large contributions of the near-bottom currents into pressure fluctuations at infrasonic frequencies. We present a machine learning-based approach, which exploits the physical properties of the flow-induced noise to infer near-bottom flow speeds from acoustic measurements at an additional deep-water location, where no current meter was available.

## Acoustic and oceanographic measurements

### Deep-water moorings

**Figure 1:**
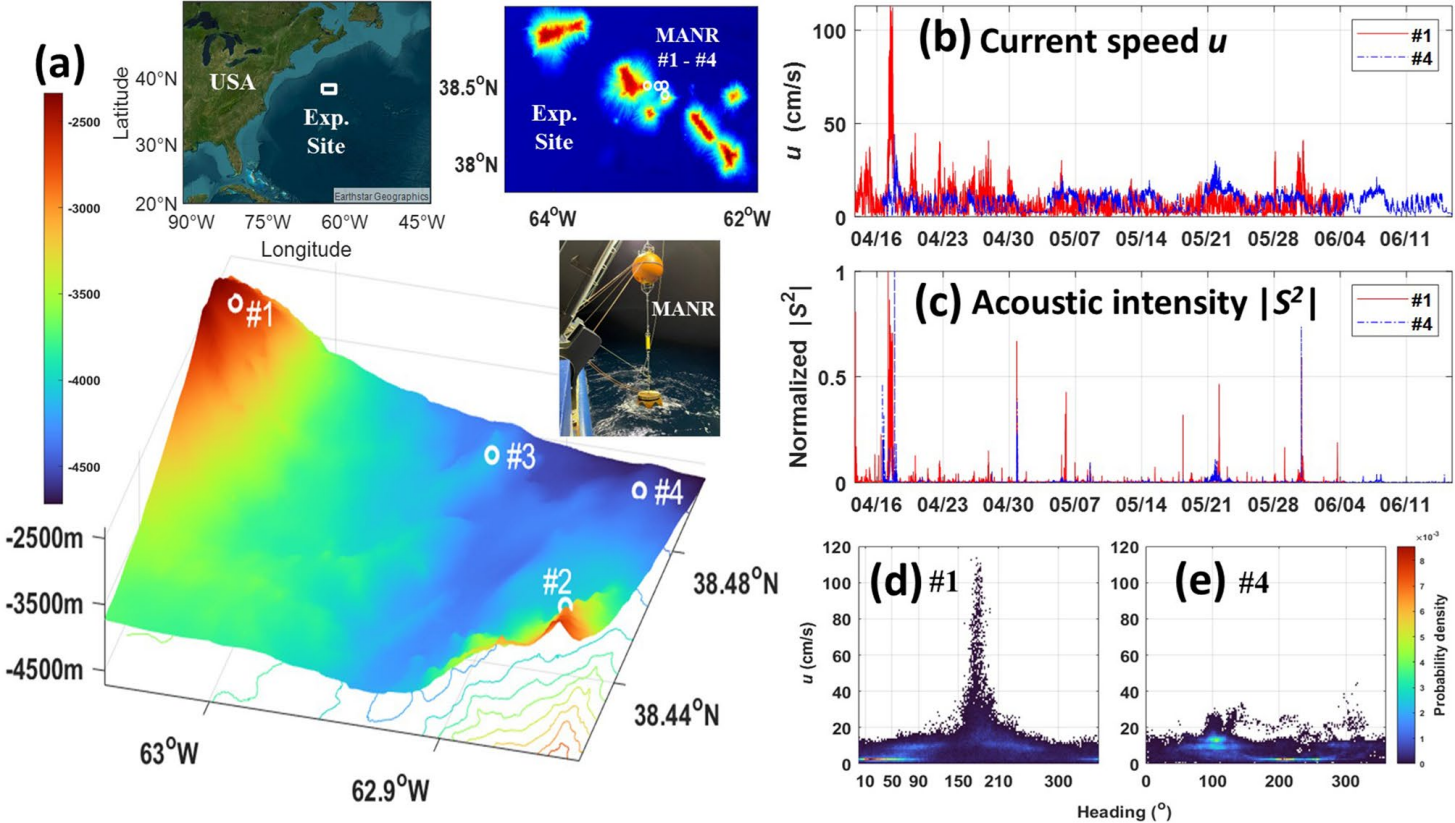
Moored Autonomous acoustic Noise Recorders (MANRs) in the 2023 NESMA Pilot Experiment. (**a**) Locations of moorings MANR#1–MANR#4. In clockwise directions, three inserts show the general geographical area of the experiment, positions of moorings and bathymetry^20^ of the experiment site on the Atlantis II seamounts, and a photograph of MANR#1 during deployment. (**b**) Time series of the flow speed measured by TCM-3 current meters on MANR#1 (red) and MANR#4 (blue) in April–June 2023. Dates and GMT time are shown on the horizontal axis. (**c**) Same as in (**b**) but for the intensity of acoustic pressure fluctuations measured by hydrophones on the same moorings. The intensity is calculated in the 1–6 Hz frequency band. For each MANR, the intensity is normalized by its maximum value during the respective observation period. (**d**) Statistical distribution of the near-bottom current velocity at MANR#1. Color shows the two-dimensional probability density function of currents with a given speed and azimuthal direction, with 0° and 90° corresponding to the currents flowing towards the North and East, respectively. (**e**) Same as in (**d**) but for currents at MANR#4.

Four Moored Autonomous acoustic Noise Recorders (MANRs) were deployed in April–June 2023 on the flanks and in the trench of the Atlantis II Seamounts (Fig. 1a). These seamounts belong to the New England Seamount Chain, which is of volcanic origin. The Atlantis II Seamounts are a group of three peaks separated by deep trenches. The individual seamounts rise from the seafloor by more than 3000 m, have steep flanks and flat tops. Depending on the dynamic position of the Gulf Stream axis, the experiment site was located in or close to the area where the Gulf Stream crosses the New England Seamount Chain and experiences strong perturbations due to interaction with the complex bathymetry^18,19^.

The primary goals of the MANR network were ambient sound and acoustic noise interferometry studies as a component of the 2023 New England Seamounts Acoustics (NESMA) Pilot Experiment. All four MANRs had identical designs, see Supplementary Fig. S1. Each MANR was equipped with a single broadband hydrophone and a chip-scale atomic clock for precision timekeeping. Acoustic pressure was recorded continuously with the sampling rate of 8 kHz. The moorings were very short (Fig. 1a and Supplementary Fig. S1), and the sensors were located about 4 m above the seafloor. One of the instruments, MANR#3, malfunctioned and did not return any data. This paper is based on the data collected by MANRs #1, #2, and #4.

MANR#1, #2, and #4 moorings were located at the points with coordinates N 38° 29.342^/^ W 63° 02.113^/^, N 38° 25.627^/^ W 62° 51.139^/^, and N 38° 29.367^/^ W 62° 51.613^/^ at distances from 7.00 to 18.13 km from each other. The deployment procedure ensured that the mooring landed on the seafloor within about 30 m from the nominal position. The water depth at the mooring locations was 2573 m (MANR#1), 2994 m (MANR#2), and 4443 m (MANR#4). Using a bathymetry database with nominal 25 m horizontal resolution^20^, bottom slopes at the mooring locations are estimated as 17.5° ± 6.2° (MANR#1), 32.5° ± 4.4° (MANR#2), and 7.1° ± 2.1° (MANR#4). The azimuthal downslope directions are 205.6° ± 17.8° at MANR#1 and 355.8° ± 20.5° at MANR#4. The slope and slope direction uncertainty is calculated as the root-mean-square (RMS) deviation from the mean in the 30 m vicinity of the nominal mooring position.

### Direct current velocity measurements

In addition to hydrophones, MANR#1 and MANR#4 were equipped with tilt current meters TCM-3 by Lowell Instruments LLC. The current meters provided point measurements with the sampling rate of 1/minute of the direction and magnitude of the horizontal velocity as well as water temperature. The vertical component of the flow velocity was not measured. On each mooring, the current meter was located about 1.5 m from the hydrophone and 4.5 m above the seafloor (Supplementary Fig. S1).

Figure 1b shows, with 1-min resolution, the results of the near-bottom flow speed measurements during the entire 52- and 60-day periods of observations by MANR#1 and MANR#4, respectively. The current speeds ranged from 0 to 1.13 m/s at MANR#1 and to 0.44 m/s at MANR#4. In contrast to the peak speeds, the average speeds, 0.0753 m/s at MANR#1 and 0.0757 m/s at MANR#4, were very similar at the two sites.

Statistical distribution of the magnitude and direction of the near-bottom flow velocity is illustrated in Fig. 1d, e. At MANR#1, the probability density function (PDF) of the near-bottom current speed peaks sharply around 0.025 m/s and has a secondary, broader peak around 0.09 m/s. The typical slow currents had a north-east direction, with a peak of the two-dimensional statistical distribution around the 20° azimuth (Fig. 1d). The moderate currents with ~ 0.09 m/s speed had even broader angular distribution with most typical directions been toward east and south-west (Fig. 1d). Strong near-bottom currents with speed over 0.20 m/s occurred about 5% of the time. The strong currents had predominantly southerly direction. Figure 1d shows that the stronger the current the tighter its angular distribution around 180°. The average current velocity at MANR#1 had 0.027 m/s magnitude and 160° direction. The average velocity made an angle of about 46° with the estimated downslope direction. At MANR#4, the more probable flow directions were around 100° and, for slow currents, in a broader range around 200°. The average current velocity had 109° direction and 0.042 m/s magnitude. The direction was somewhat close to but different from the estimated isobath direction. The magnitude of the average current velocity proves to be significantly larger than at MANR#1 due to a tighter directional distribution of the near-bottom velocities.

**Figure 2:**
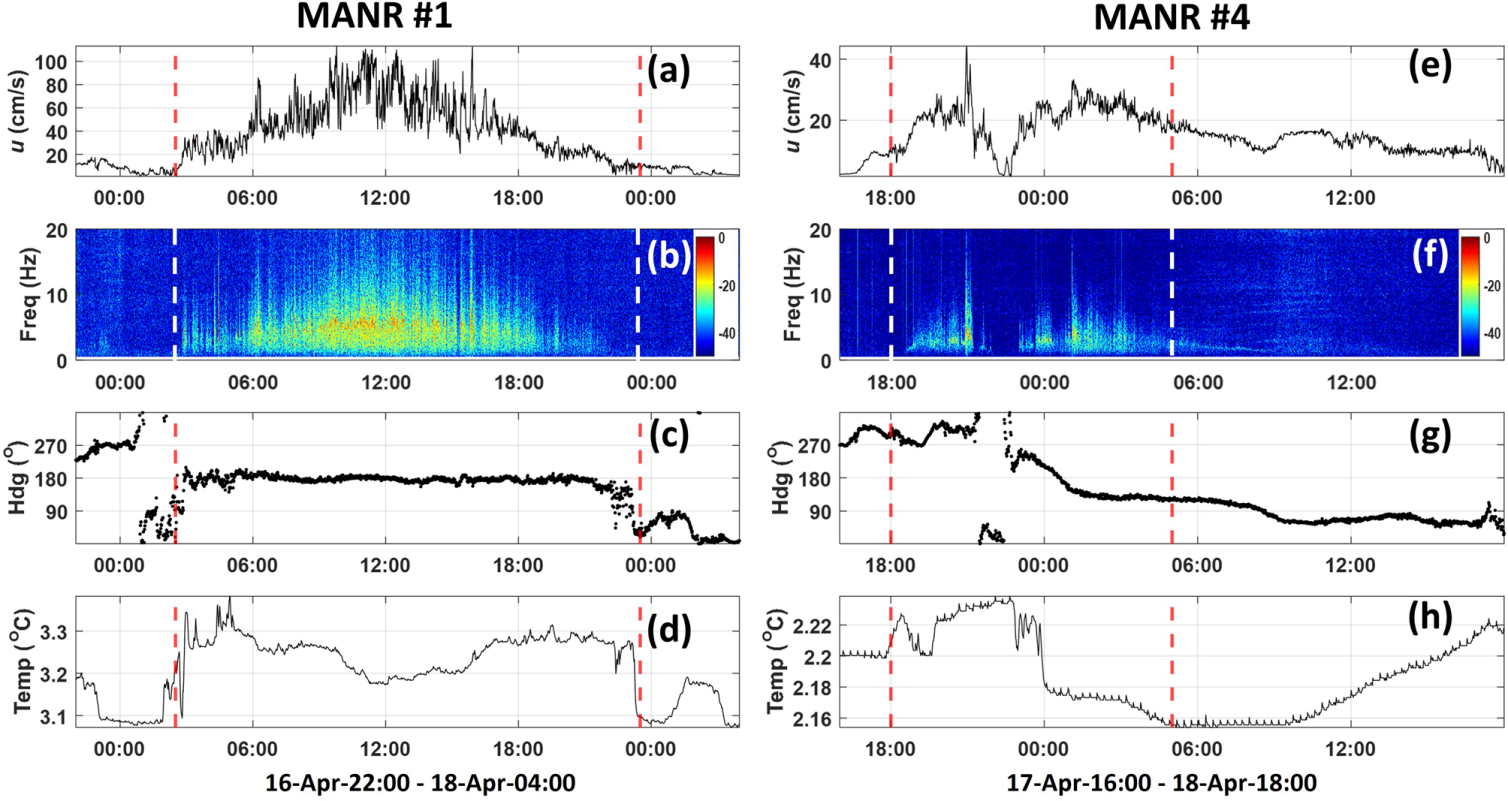
Temporal variation of the near-bottom flow velocity, water temperature, and low-frequency acoustic noise during strong-current events. (**a**) Current speed, (**b**) spectrogram of acoustic pressure, (**c**) direction of the horizontal flow, and (**d**) water temperature at MANR#1 measured on 16–18 April 2023 in the 30-h period starting at 22:00:00 on April 16. Vertical dashed lines delineate the 21-h period from 02:30–23:30 on April 17 when the strongest currents were observed. GMT time is shown on the horizontal axis in hours. (**e–h**) are the same as (**a–d**), respectively, but for measurements made at MANR#4 on 17–18 April 2023 in the 26-h period starting at 16:00:00 on April 17. Vertical dashed lines delineate the 11-h period from 18:00 on April 17 to 05:00 on April 18 when the strongest currents were observed at MANR#4. The acoustic power spectrum density is shown in (**b, f**) in dB relative to its maximum value in each figure.

Observations of the strongest current events at MANR#1 and MANR#4 are illustrated in Fig. 2a–d, e–h, respectively. Extreme currents at MANR#4 were observed with about 14 h delay from MANR#1. At both sites, increases in the flow speed were clearly accompanied by increases in flow noise intensity and frequency (cf. Fig. 2a, b, e, f). The beginning and end of extreme current event at MANR#1 were accompanied by rapid temperature increase and drop, respectively, by about 0.2 °C (Fig. 2d). During the entire 21-h extreme event the flow direction remained remarkably stable at MANR#1 and was drastically different from the directions before and after the event (Fig. 2c). The flow direction was close to but different from the estimated downslope direction. All these distinctive features were reproduced, albeit not quite as clearly, in other strong currents events at MANR#1, see Supplementary Fig. S2.

Aside from not being as strong, the extreme current events at the deeper MANR#4 site may have different characteristics than at MANR#1. The strongest currents neither maintained a constant direction nor elevated water temperature for as long as at MANR#1 (Fig. 2g, h). Features of the strong current events at MANR#4 may also be less consistent than at MANR#1. An example of a current event at MANR#4 with the peak speed of 0.30 m/s, which is illustrated in Supplementary Fig. S3, suggests an elevated temperature and constant flow direction accompanying fast near-bottom flows. This is qualitatively similar to the extreme current events at MANR#1.

### Flow noise

Turbulent flows are associated with particle velocity and pressure fluctuations (pulsations) that evolve slowly in the reference frame moving with the mean flow^21^. When the turbulent pulsations are advected by the flow past a hydrophone, the pressure fluctuations are recorded at the apparent frequency *u/L*, where *u* is the mean flow velocity and *L* is a representative spatial scale of the pressure fluctuation. These pressure fluctuations do not represent any sound waves and are known as pseudo-sound^22^. For hydrophones located at or near the seafloor, both the inherent turbulence of the boundary layer flow and the vortex shedding caused by an obstacle (the mooring) in the flow contribute to the flow noise. The observed noise spectrum is further affected by dimensions and geometry of the hydrophone due to averaging of the pressure fluctuations over its surface^21^.

The pressure fluctuations recorded by each MANR include the contributions of the compressional waves (sound) and of the local flow (pseudo-sound). For brevity, we will refer to the pressure fluctuations detected by a hydrophone and their intensity in the infrasonic frequency band as acoustic pressure and acoustic intensity regardless of the physical nature of the fluctuations. The contributions of both sound and pseudo-sound to the acoustic intensity are highly variable and appear random. They cannot be separated on a single-hydrophone receiving system such as a MANR without additional data. However, the sound intensity and spectrum are uncorrelated with the local current velocity, while the pseudo-sound (flow noise) spectrum expands to higher frequencies and intensity increases, on average, with the current speed^22^. Thus, the measured acoustic intensity contains information about the current speed.

Flow noise is found to make a large and often dominant contribution to pressure fluctuations at MANRs. A visual inspection of Fig. 1b, c shows strong similarity of the current speed and acoustic intensity time series at the same MANR. For each MANR, peaks of the current speed and intensity appear at the same time, and higher current speed peaks tend to correspond to higher intensities. At MANR#1, the current speed and acoustic intensity prove to be significantly correlated during the entire observation period, which includes episodes of swift currents and longer periods of slow currents. The Pearson correlation coefficient of the current speed squared and the acoustic intensity time series in Fig. 1b, c is 0.873 for MANR#1.

**Figure 3:**
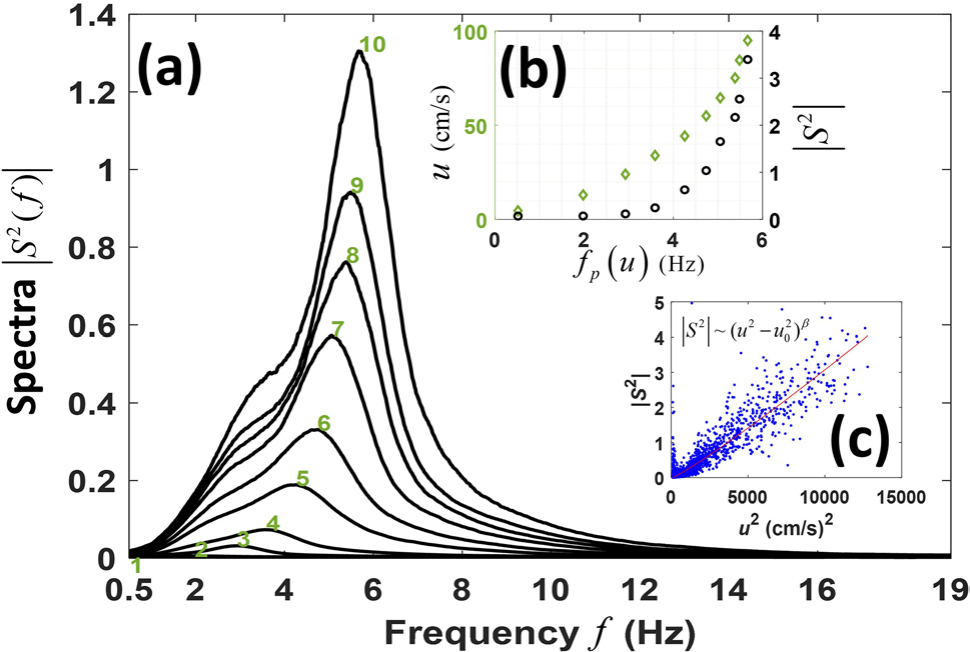
Flow noise spectra and intensity at different current speeds at MANR#1. (**a**) Average spectra of low-frequency noise are shown for current speeds in 0.10 m/s intervals centered at 0.05 m/s (line 1), 0.15 m/s (2), 0.25 m/s (3), …, 0.95 m/s (10). Spectral densities at any frequency *f* are calculated as acoustic intensity in the 1 Hz frequency band centered on *f* and are averaged over all observations with current speeds in the respective range. The spectral densities are referenced to 8.9125∙10^9^ μPa^2^/Hz. (**b**) Relation between the peak frequency, where the averaged flow noise spectrum is maximum, and current speed and the noise intensity is shown using the left and right vertical axes, respectively. The current speed is shown by green diamonds. The noise intensity is calculated in the 0.5–19 Hz frequency band and is shown by open black circles. (**c**) Relation between the current speed and intensity of low-frequency noise in the 1–7 Hz frequency band in individual measurements (with 1-min averaging) is shown with blue dots for the entire observation period. Only observations with current speeds over 0.10 m/s are shown. The red line illustrates the optimum least-squares regression for the dependence of the noise intensity |*S*^2^| on the current speed *u* at *u* > 0.10 m/s. Parameters of the regression are given in the text. In (**b, c**), the intensity is referenced to the intensity of a signal with root-mean-square pressure of 0.09441 Pa.

Results of a detailed investigation of spectral properties of the observed low-frequency noise are illustrated in Fig. 3. Pressure fluctuations were analyzed in the infrasonic frequency band 0.5–20 Hz. After averaging over observations with similar values of the current speed to suppress random variations, acoustic intensity is found to increase steadily with the current speed at all frequencies (Fig. 3a). In Fig. 3a, the measurements made at MANR#1, when current speeds *u* ranged from 0 to 1.00 m/s, are split in 10 groups with progressively increasing *u*. In the *jt*h group the speed is in the band 0.1(*j *− 1) m/s ≤ *u* < 0.1*j* m/s. Since the statistical distribution of the observed flow speeds is nonuniform, the average values of the speed in each of the ten speed bands deviate from the center of the band. The average current speeds within each of the bands in Fig. 3a are 0.0456, 0.1300, 0.2399, 0.3393, 0.4427, 0.5497, 0.6456, 0.7512, 0.8451, and 0.9501 m/s, respectively. The number of observations within the speed band decreases with the central speed, and the number of data points in the first and tenth band in Fig. 3a differ by three orders of magnitude. There are not enough observations with *u* > 1.00 m/s to provide reliable statistics of noise, and noise spectra for such observations are not included in Fig. 3a. A similar, but slightly less regular variation of the noise spectra with current speed is found when the observations are split into the narrower, 0.01 m/s, bands with fewer data points per band (Supplementary Fig. S4a). At MANR#1, the narrow speed bands remain sufficiently populated for a meaningful averaging only up to speeds of about 0.30 m/s.

The averaged noise spectra in Fig. 3a and Supplementary Fig. S4a have a single, well-defined maximum. Increase in the power spectral density of noise with the current speed is accompanied by a shift of the peak frequency *f*_*p*_, where the spectrum is maximum, towards higher frequencies (Fig. 3a and Supplementary Fig. S4a). Variations of the noise intensity and peak frequency with current speed are summarized in Fig. 3b in terms of the average spectra for the current bands that are shown in Fig. 3a. The trends in both intensity and peak frequency in Fig. 3b are consistent with expectations for flow (or flow-dominated) noise^9,11,22^.

To obtain a quantitative empirical dependence of flow noise intensity on the current speed, we used an ensemble of MANR#1 measurements in non-overlapping one-minute time windows (Fig. 3c). Assuming the dependence $\left| {S^{2} } \right| = const. \cdot \left( {u^{2} - u_{0}^{2} } \right)^{\beta }$ between flow-noise intensity and current speed *u* at *u* > 0.10 m/s, a least-squares regression of the measured intensities in the 1–7 Hz band gives *β* = 1.10 ± 0.01, *u*_0_ = (0.097 ± 0.003) m/s. The uncertainty in the exponent *β* and *u*_0_ are determined at the 95% confidence level. Observations with the speed of at least 0.10 m/s are chosen as the data. The frequency band and the lower bound of the flow speed are selected here to minimize the non-flow-noise contributions into the noise intensity.

With the dependence of the flow noise intensity established, insights into the role of flow and non-flow noise can be obtained by extending the scatter plot in Fig. 3c to all one-minute measurements (Supplementary Fig. S4b). Individual data points form two clusters. At *u* < 0.10 m/s, a full range of intensities, up to the maximum observed intensity, has been observed due to contributions of distant acoustic sources. The other cluster of data points is formed around the established empirical relation between noise intensity and current speed and represents flow-noise dominated measurements. The two groups of data points are clearly separated except when both the intensity and current speed are relatively small (Supplementary Fig. S4b).

Distinctions between flow and non-flow noise are manifested in their spectral characteristics. It is illustrated in Supplementary Fig. S4c in terms of dependence between the centroid frequency *f*_*c*_ of noise and the current speed at MANR#1. The centroid frequency characterizes the shape of the power spectrum and is calculated for each one-minute measurement as the frequency weighed by the noise power spectral density. For non-flow noise, *f*_*c*_ can take any value in the 0.5–20 Hz signal processing frequency band regardless of the current speed *u*, while the flow noise-dominated measurements form a tight cluster with *f*_*c*_ steadily increasing with *u* and reaching about 6 Hz for the strongest currents. The two groups of data points overlap, when both *f*_*c*_ and *u* are relatively small, and become separated for stronger currents.

### Ocean current speed inference from the flow noise data

In underwater acoustics, flow noise is often viewed as a nuisance and an obstacle for target detection or characterizing the effects of anthropogenic activity on marine life^9,10,12^. Here, we take the opposite view of flow noise as a source of information about the flow that created the observed noise. We employ judiciously chosen spectral characteristics of the observed pressure fluctuations to infer the near-bottom current speed.

The acoustic and current velocity data acquired by MANR#1 are employed to develop a supervised machine learning regression^23,24^ of noise spectrograms to predict the current speed. Time series of the near-bottom flow speed with 1 min temporal resolution is derived by a neural network (NN)^23,24^ from concurrent measurements of pressure fluctuations. Initially, the shape of the power spectrum $\left| {S^{2} (f)} \right|$ in the 0.5–19 Hz frequency band served as input for the NN model. Intensity in each frequency bin was centered and scaled by the corresponding mean and standard deviation. However, this initial model, when applied to MANR#2 data, occasionally produced unrealistically strong currents when the measured noise intensity was low, which contradicted the flow noise properties established in the previous section and illustrated in Fig. 3c. The cause of this discrepancy was identified as overfitting the model to training data. To address this issue, two integral quantities, the total intensity $\left| {S^{2} } \right|$ in the infrasonic band and the centroid frequency *f*_c_, were introduced as additional inputs. To ensure the comparable weighting of two extra quantities with the other inputs, Principal Component Analysis (PCA)^22,24^ was used to reduce feature dimensions and mitigate overfitting. 95% of the total data variance was retained after PCA feature transformation.

**Figure 4:**
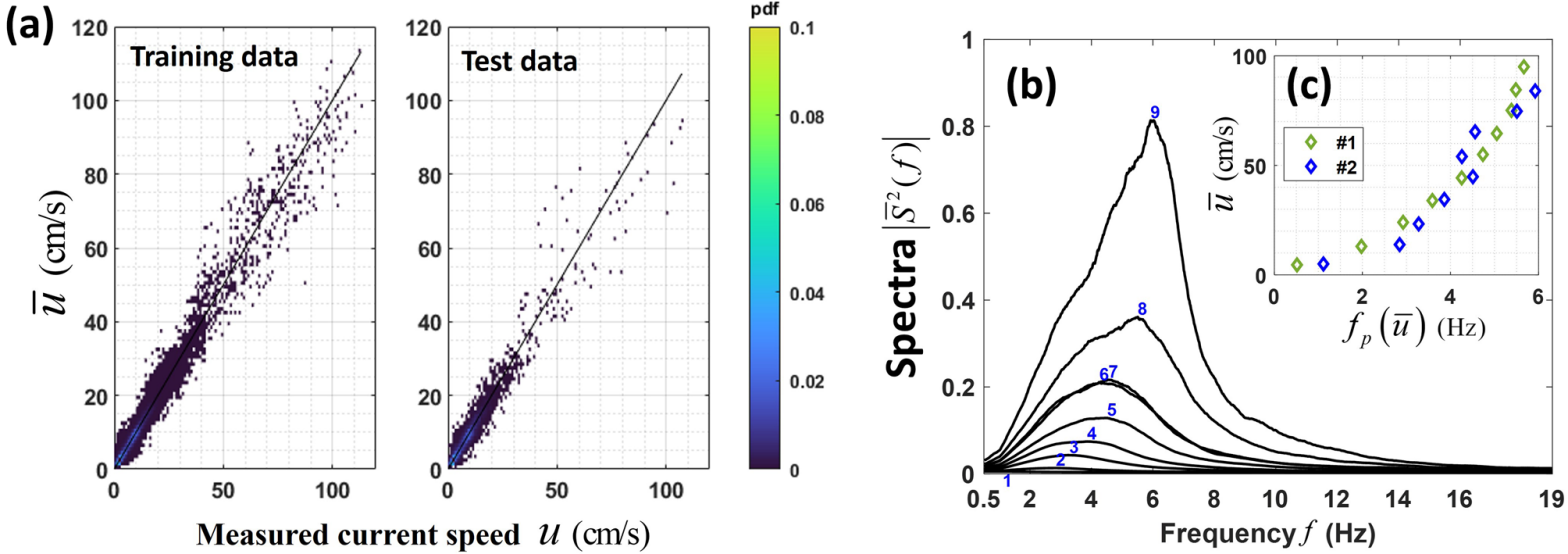
Neural network (NN) model to infer current velocity from flow noise measurements. (**a**) Relation between the measured and NN-predicted current speed in the training (left) and testing (right) datasets. Each dot represents one minute of noise and current measurements at MANR#1. Density of the point distribution is shown with color scale. 80% and 20% of the MANR#1 data are used for training and testing, respectively. RMS deviations of the NN predictions from the current speed measurements are 0.014 and 0.019 m/s in the training and testing datasets, respectively. (**b**) Same in Fig. 3a but for noise spectra measured at MANR#2 and the flow speed at MANR#2 inferred using the NN model. (**c**) Relation between the peak frequency, where the averaged flow noise spectrum is maximum, and current speed directly measured at MANR#1 (green diamonds) or inferred at MANR#2 using the NN model (blue diamonds).

With the acoustic data after the PCA-based feature reduction as the input, the feed-forward NN employed for the flow speed inference comprised five hidden layers. The input and output layers had 100 nodes each; each hidden layer had 80 nodes. The Rectified Linear Unit (ReLU) was used as the activation function^23^. Randomly selected 80% of MANR#1 data, or 41.5 days’ worth of continuous measurements, served as the training dataset and the other 20%, or 10.5-days’ worth of measurements, were reserved for testing. Results of the NN training and testing are illustrated in Fig. 4a. The RMS deviation between the NN flow speed model $\overline{u}$ and direct measurements *u* was 0.014 m/s and 0.019 m/s for the training and testing data sets, respectively. The model-data fit is excellent for *u* < 0.50 m/s, where most data points reside. The data-model discrepancy is larger at higher flow speeds (Fig. 4a). This can be attributed, first, to wider spread of intensities around the mean (see Fig. 3c) and, second, fewer data points available at higher current speeds. The near-bottom flow speeds *u* > 0.50 m/s were encountered at MANR#1 for only about 12 h during the entire observation period, or about 1% of the time (Figs. 1b and 2a).

**Figure 5:**
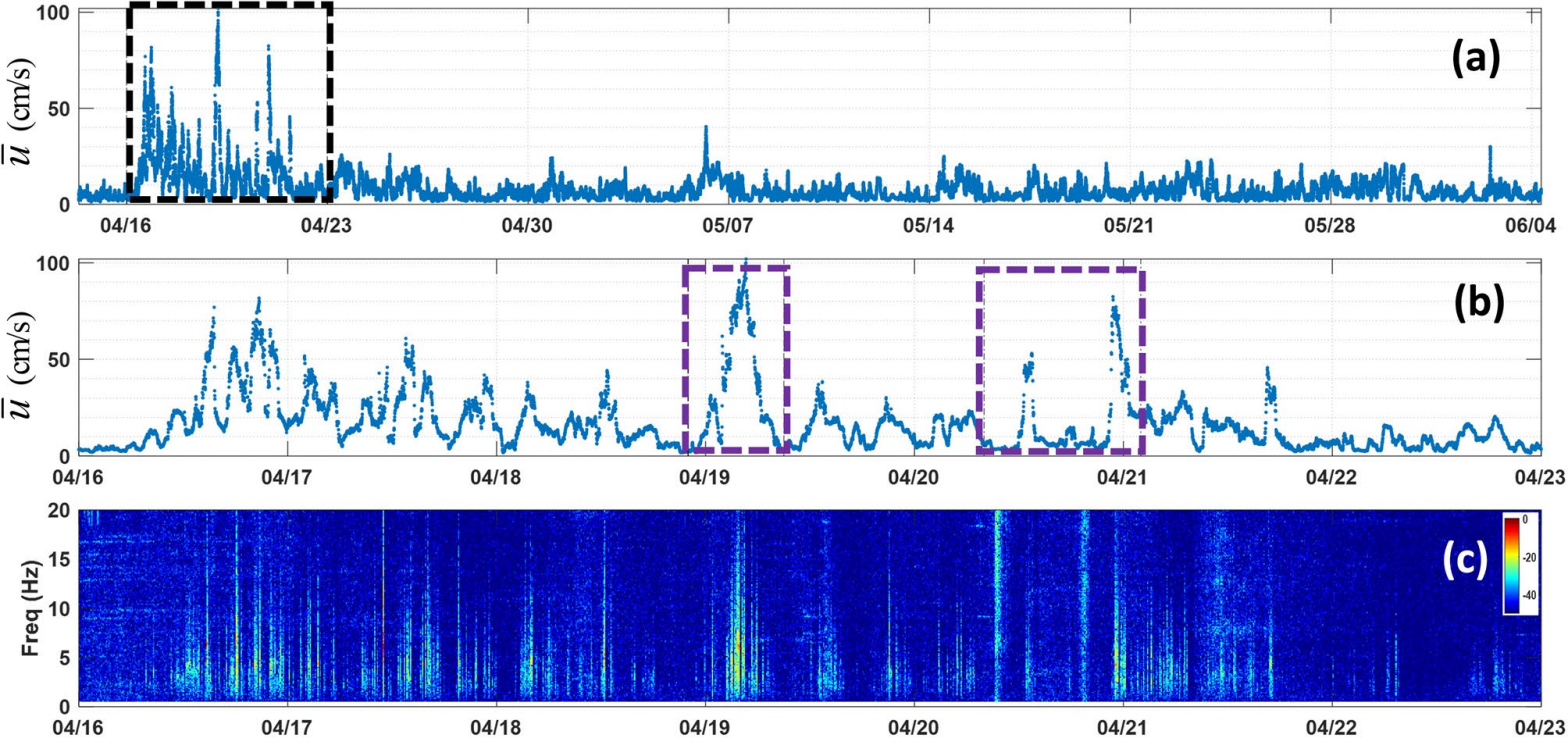
Near-bottom current speed and flow noise at MANR#2. (**a**) Current speed $\overline{u}$ inferred by the NN model from the pressure fluctuation measurements at MANR#2 during the entire period of the mooring deployment from 14 April to 4 June 2023. (**b**) Expanded view of the inferred currents during a week-long period indicated in (**a**) by a dashed box. Two purple dashed boxes indicate time windows shown in greater detail in Fig. 6. (**c**) Spectrogram of pressure fluctuations during the same period as in (**b**). The acoustic power spectrum density is shown in dB relative to its maximum value in the figure.

The NN model was applied to acoustic data acquired at MANR#2 and has produced the time series of the near-bottom current speed shown in Fig. 5a. Figure 4b, c show the average flow noise spectra at MANR#2 and their peak frequency that are obtained by combining the inferred speeds $\overline{u}$ and pressure fluctuations measurements. As with the directly measured current speeds at MANR#1, statistical distribution of the inferred speeds at MANR#2 is nonuniform. Therefore, the average values of the speed in each of the speed bands illustrated in Fig. 4b deviate from the center of the band. The average current speeds within the bands 1–9 in Fig. 4b are 0.0498, 0.1382, 0. 2326, 0.3447, 0.4481, 0.5403, 0.6534, 0.7472, and 0.8400 m/s, respectively. These values are close to but differ from the average speeds in the respective bands in Fig. 3a. Of the total number of 73,437 one-minute measurements at MANR#2, only 45 contribute to calculation of the average spectra in the ninth band in Fig. 4b, and there are not enough observations for calculation of a reliable average spectra at the speeds exceeding 0.90 m/s. The observations corresponding to *u* > 0.90 m/s are not included in Fig. 4b. The flow speed and frequency dependences of the spectra in Fig. 4b are qualitatively and, except for lines 7 and 8, quantitatively similar to the MANR#1 spectra (Fig. 3a), which are obtained using direct flow speed measurements. The spectral magnitudes in the 0.60–0.80 m/s speed range (lines 7 and 8) appear to be underestimated. This is probably related to the NN model testing data (Fig. 4a) showing a broader spread of $\overline{u}$ around true current speeds *u* in this range.

The dependencies of the peak frequencies on the flow speed are consistent at MANR#1 and MANR#2 (Fig. 4c) and in fact between all three MANRs, see Supplementary Fig. S5a. Dependencies between the flow noise centroid frequency *f*_*c*_ and intensity at MANRs 1 and 2 are also consistent (Supplementary Fig. S5b). The agreement between the flow noise properties at MANR#1 and MANR#2 justifies application of the NN model to MANR#2 acoustic data.

**Figure 6:**
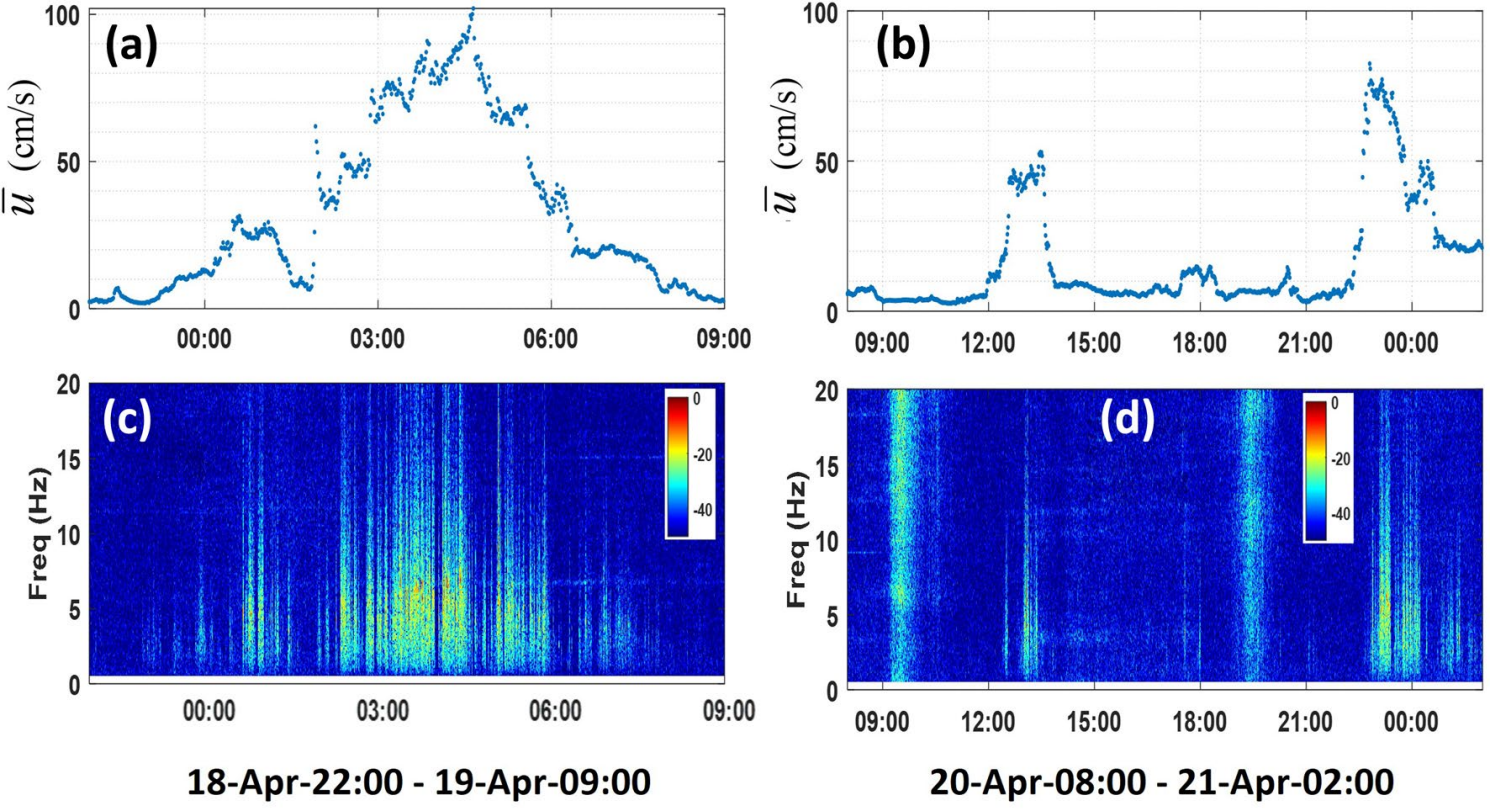
Inferred near-bottom current speed and measured low-frequency acoustic noise at MANR#2 during two events. (**a**) Current speed inferred by the NN model during a 12-h period on 18–19 April 2023. (**b**) Current speed inferred by the NN model during an 18-h period on 20–21 April 2023. (**c**) Spectrogram of pressure fluctuations during the same period as in (**a**). (**d**) Spectrogram of pressure fluctuations during the same period as in (**b**). The acoustic power spectrum density in the spectrograms is shown in dB relative to its maximum value in each figure.

Several strong-current episodes are inferred to occur at MANR#2 with the maximum near-bottom speed of up to 1.00 m/s (Fig. 5a). The mean near-bottom flow speed was 0.0808 m/s. Temporal variations of the current speed and noise spectra are shown in more detail in Fig. 5b, c for the seven-day period, which encompasses the strongest current events. Near-bottom flow speed exceeded 0.50 m/s on nine occasions during this period. Two shorter segments within this time frame are enlarged further in Fig. 6. The strong-current episodes at MANR#2 were comparable in magnitude and duration to those at MANR#1 (cf. Figures 5a, b, and 6a with Figs. 1b and 2a). The strong-current events did not occur simultaneously at the two sites and appear to be separated by 12–14 h.

As expected, noise intensities are high, and spectrograms are similar with spectral maxima below 10 Hz during the strongest current events at MANR#1 (Fig. 2a, b) and MANR#2 (Fig. 6a, c). The same behavior can be seen in Fig. 6b, d between 22:00 on April 20 and 01:00 on April 21, when $\overline{u}$ reaches 0.80 m/s. Noise intensity is equally strong between 09:30–10:00 and 19:00–19:30 on April 20. However, it is clear to a human analyst from the spectrum, which neither decreases at higher infrasonic frequencies nor exhibits the characteristic temporal intermittency, that these pressure fluctuations are not flow noise. The NN model correctly interprets these events and predicts modest flow speeds (Fig. 6b, d).

## Discussion

The current speeds at MANR#1 cover a much broader range of values than at the deeper MANR#4 site. In addition, since flow noise intensity increases rapidly with flow velocity, MANR#1 data are expected to have a considerably larger number of samples, where flow noise provides a dominant contribution to intensity of low-frequency pressure fluctuations, than MANR#4 data. Therefore, we relied solely on MANR#1 data in determining the empirical dependence of the flow noise intensity on current speed, as described above (Fig. 3). That also helped to avoid the biases that would be caused by a possible difference in the MANR#1 and #4 hydrophone sensitivities.

The similarity of the water depths at the MANR#1 and MANR#2 sites and their locations on steep seamount flanks made MANR#1 (as opposed to MANR#4) current measurements and acoustic data a natural choice for developing a model to infer MANR#2 current speeds from flow noise spectra. The maximum current speeds found at MANR#2 are several times larger than at MANR#4 and almost as large as at MANR#1 (cf. Figs. 1b and 5a). That confirms retrospectively that MANR#4 acoustic data, although consistent with MANR#1 data in the common range of flow speeds (Supplementary Fig. S5a), would not have sufficient diversity of the noise spectra to train a neural network capable of handling MANR#2 acoustic data.

It is instructive to put results of this study in the context of previous observational evidence and numerical simulations of extreme near-bottom currents in deep ocean. Oliver et al*.*^13^ modeled extreme near-bottom currents across the northwest Atlantic, including the New England Seamount Chain. No currents with extreme speeds exceeding 0.7 m/s were predicted to occur with either 17- or 50-year return period at depth over 1700 m. Our results indicate that actual near-bottom currents can far exceed the published estimates^13^ of the maximum current speeds.

At various deep-water sites, seabed photography and acoustic data reveal the diverse bedforms and other current-controlled seabed features that require at least episodic current events with maximum speeds of almost 1.0 m/s or faster^1,4,5,25^. To our knowledge, such speeds were not previously directly measured or predicted by circulation models at depths of over 2000 m. Results of this study narrow the gap between oceanographic and geomorphological observational evidence of the extreme near-bottom current speeds in deep ocean. At the same time, our results indicate that a further improvement of ocean circulation models is needed to match observations of the extreme current events.

The stronger current events with *u* ≥ 0.40 m/s occurred between April 16 and 22 at all three MANR sites. Identifying the specific dynamic process or processes responsible for the extreme current events observed in the NESMA experiment is beyond the scope of this paper and may require additional data. We note only that other benthic storms in the same general area of the northwest Atlantic were attributed to the Deep Western Boundary Current^1,4,15,25^ and deep cyclogenesis^2,3,17,26,27^. In particular, strong deep currents are expected to be created by cyclogenesis under a trough of a Gulf Stream meander, with mixed barotropic-baroclinic instability playing a key part in the process^17^. The latter explanation appears more probable for the extreme current events at MANR#1 and MANR#2 given the proximity of the experiment site to the Gulf Stream. The explanation is further amplified by the prediction that occurrence of strong near-bottom currents due to a topographically generated eddy would be accompanied by a warm anomaly on the slopes of the seamount^26^ as in our observations (see Fig. 2d). However, it is not immediately clear how to reconcile the hypothetical cyclonic origin of the near-bottom flow with the current velocity maintaining a constant southward direction (Fig. 2c) during the entire extreme current event.

## Conclusions

Extremely strong near-bottom flows have been observed at three deep-water locations in the New England Seamounts, including sustained currents with speed over 1.10 m/s at 2573 m depth. This is the fastest current speed ever directly measured in the ocean at a comparable or larger depth. The extreme currents occur as episodes with duration of the order of 10 h and were observed repeatedly over 51–60-day deployments. The strongest currents had a consistent direction close to the downslope direction on a steep seamount flank and were accompanied by a distinctive increase in the water temperature.

Direct measurements of extreme flow velocities are confirmed by collocated acoustic measurements. Strong correlation is found between the current speed and the intensity and spectral properties of pressure fluctuations in the infrasonic frequency band. Having trained a neural network on data from a site where collocated flow velocity and acoustic measurements were done, flow noise spectra were inverted for near-bottom current speed by the neural network model at an additional site where only acoustic data were acquired on an identical mooring.

Our observations of the extreme near-bottom abyssal currents with speeds over 1.10 m/s help to close the gap between geological evidence for extreme near-bottom currents and the direct oceanographic measurements of the current speed. The likelihood of the occurrence of extreme current events should be considered when designing scientific moorings and long-term underwater infrastructure. To our knowledge, the maximum observed speeds exceed available model predictions and thus suggest that ocean circulation models may require higher spatial resolution and probably other improvements to reproduce the observations.

Further research is needed to characterize the frequency of occurrence and geographical distribution of the extreme abyssal current events described in this paper and unambiguously identify the ocean processes creating the extreme near-bottom currents.

## Methods

### Analysis of bathymetry

The magnitude and the azimuthal direction of the bottom slope at locations of the moorings were calculated from the cubic spline interpolation of the bathymetry dataset^20^, which has nominal 25 m horizontal resolution. Uncertainty in the bottom slope’s azimuthal direction and magnitude were calculated as the root-mean-square deviations of the azimuthal angle and the angle between the normal to the seafloor and the vertical, respectively, from the mean values of these angles in the 30 m vicinity of the nominal position of the mooring.

### Tilt current meter data

Direct measurements of the flow speed by TCM-3 current meters were screened for outliers. Outliers were defined as points in the time series where a measurement exceeded by more than 50% and more than 0.05 m/s the half-some of the measurements at the immediately preceding and following points, i.e., within 1 min of the measurement in question. The outliers were removed and replaced by the mean of the immediately preceding and following measurements. The outliers accounted for 0.23% of the total number of points for MANR#1 and less than 0.001% for MANR#4.

The resolution of TCM-3 temperature measurements is 0.001°C and is responsible for the 0.001°C jitter that is visible in Fig. 2h.

### Hydrophone data analysis

MANR#1, MANR#2, and MANR#4, hydrophones had sensitivities of − 177.9, − 178.0, and − 177.8 dB re 1 V/µPa, respectively, and a factory-set preamplifier gain of 18.4 dB. Only relative values of the acoustic intensity are used in this study, and therefore the results are unaffected by possible deviations of hydrophone sensitivities and preamplifier gains from their nominal values provided by the manufacturer. The spectra of pressure fluctuations that are shown in the spectrograms were obtained for each non-overlapping one-minute time interval of hydrophone data. The spectra were calculated in the 0.5–20 Hz band at 1/60 Hz steps as a moving average of the intensity of pressure fluctuations in a 1 Hz frequency band. Narrower bands were used for averaging when the central frequencies were below 1.0 Hz or above 19.5 Hz.

Estimation of the centroid frequency *f*_*c*_ of noise in the infrasonic frequency band is based on calculation of the power spectra of Hamming-windowed, two-second-long segments of data with 1.75 s overlap, and subsequent averaging of the result within each one-minute-long observation period.

### Supplementary Information

Supplementary Information.

## Data Availability

The datasets generated during the current study are available from the corresponding author on reasonable request.
