# Supplementary material for: Observation of exceptionally strong near-bottom flows over the Atlantis II Seamounts in the northwest Atlantic
Source: Sci Rep. 2024 May 5;14:10308. doi: 10.1038/s41598-024-60528-2 (PMC11070428; doi:10.1038/s41598-024-60528-2)
Supplement: Supplementary file 1 — Supplementary Information. [file 41598_2024_60528_MOESM1_ESM.pdf]

Observation of exceptionally strong near-bottom flows over the Atlantis II Seamounts in the northwest Atlantic

Supplementary information

Oleg A. Godin[1, *], Tsu Wei Tan[2], John E. Joseph[3] & Matthew W. Walters[1]

[1] Physics Department, Naval Postgraduate School, Monterey, CA 93943, USA

[2] Department of Marine Science, ROC Naval Academy, Kaohsiung 81345, Taiwan

[3] Oceanography Department, Naval Postgraduate School, Monterey, CA 93943, USA

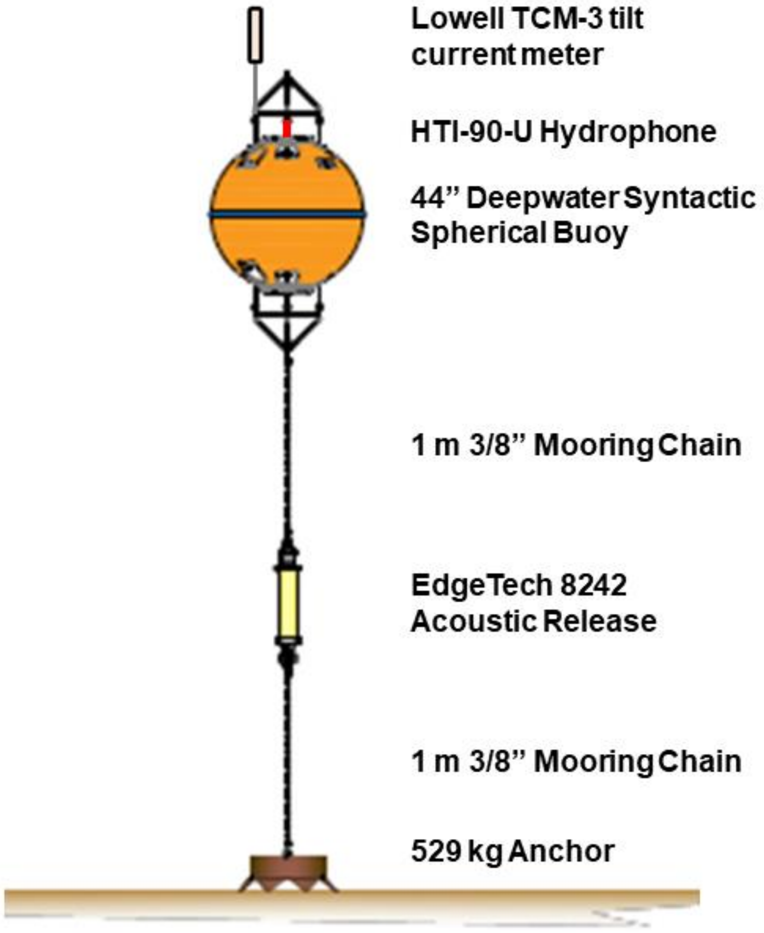

Supplementary Figure S1. Moored Autonomous acoustic Noise Recorder (MANR).

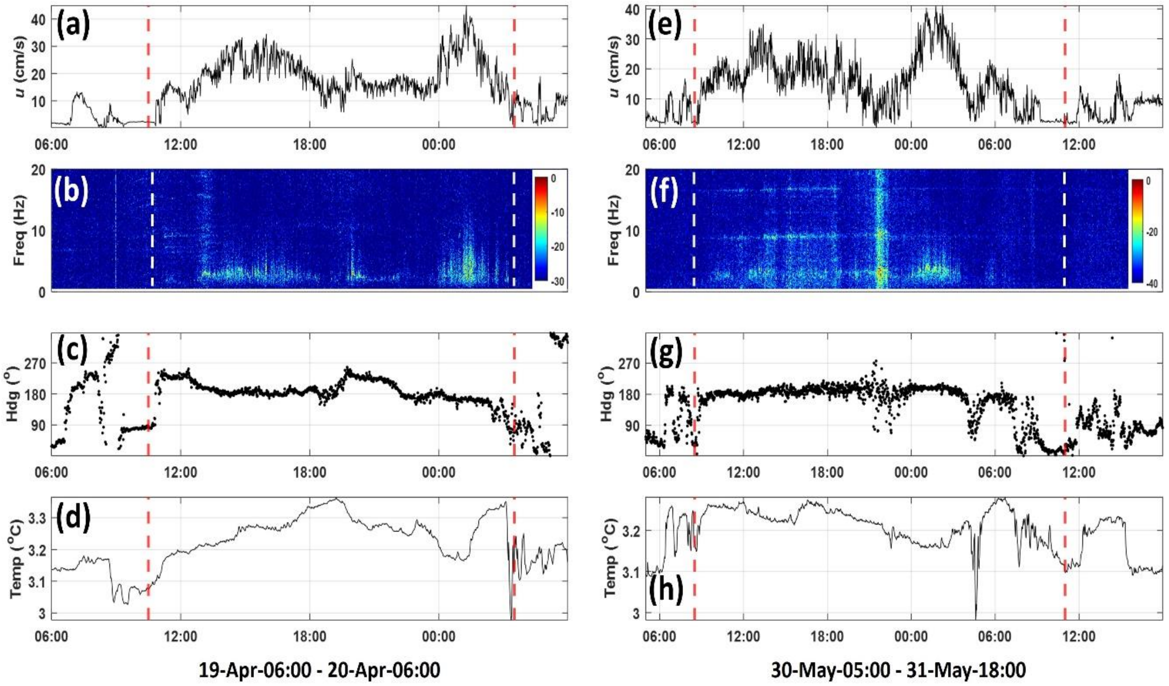

Supplementary Figure S2. Observed variations of oceanographic and acoustic parameters during selected strong-current events at the MANR#1 site. **(a)** Near-bottom current speed, **(b)** spectrogram of acoustic pressure, **(c)** direction of the horizontal flow, and **(d)** water temperature measured in the 24-hour period starting at 06:00:00 on 19 April 2023. Vertical dashed lines delineate a 17-hour period when the stronger currents were observed. GMT time is shown on the horizontal axis in hours. Acoustic power spectrum density is shown in dB relative to its maximum level during the observation period. **(e)**, **(f)**, **(g)**, and **(h)** are the same as (a), (b), (c), and (d), respectively, but for measurements made in the 37-hour period starting at 05:00:00 on 30 May 2023. Vertical dashed lines delineate the 26.5-hour period when the stronger currents were observed. The acoustic power spectrum density is shown in figures (b) and (f) in dB relative to its maximum value in each figure.

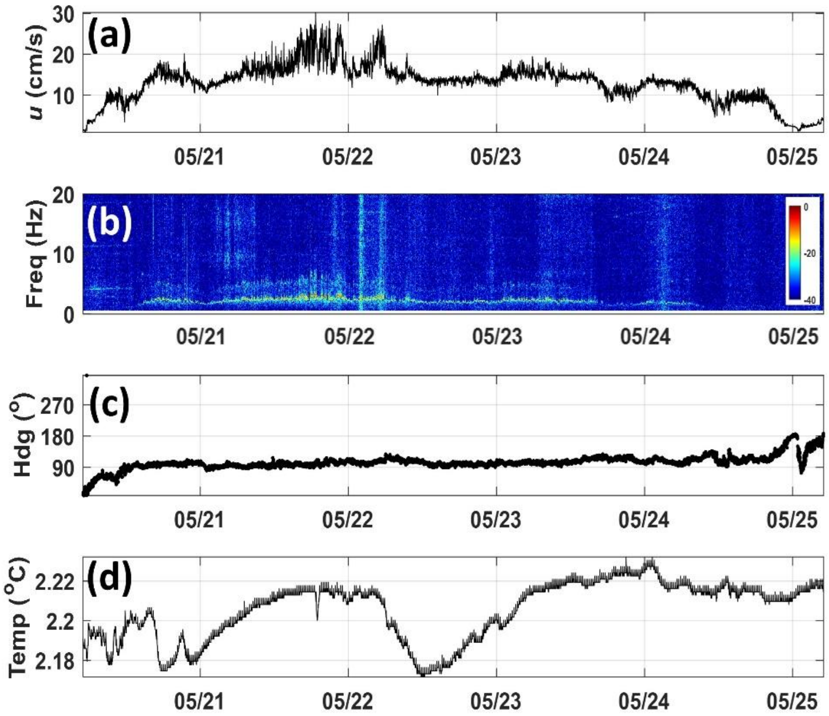

Supplementary Figure S3. Observed variations of oceanographic and acoustic parameters during a strong-current event at the MANR#4 site. **(a)** Near-bottom current speed, **(b)** spectrogram of acoustic pressure, **(c)** direction of the horizontal flow, and **(d)** water temperature measured in the 120-hour period starting at 05:00:00 on 20 May 2023. GMT time and dates are shown on the horizontal axis. The acoustic power spectrum density is shown in figure (b) in dB relative to its maximum value in the figure.

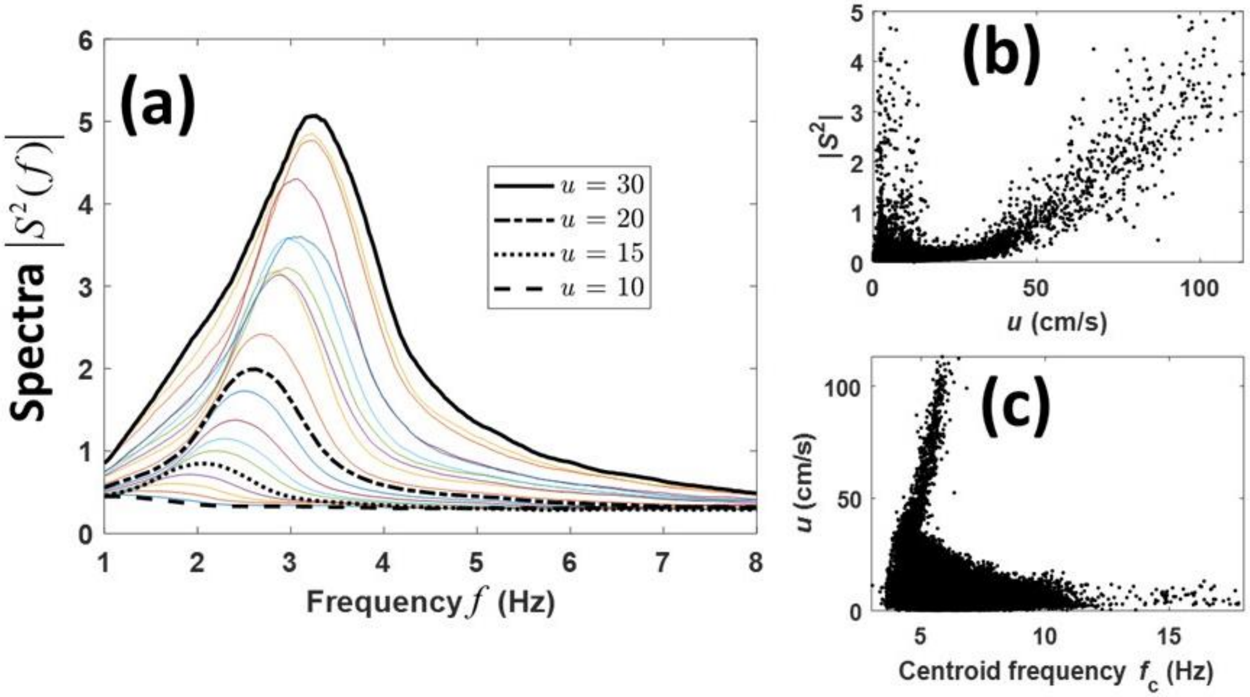

Supplementary Figure S4. Physical properties of the flow noise observed at MANR#1. **(a)** Spectra of pressure fluctuations that are observed at different current speeds are shown for the current speeds $\tilde{u}$ from 10 to 30 cm/s in 1 cm/s intervals. Spectra are calculated as a moving average in 1 Hz frequency band and are further averaged over all observations with current speeds u in the interval $\tilde{u} - 0.5\,\text{cm/s} \le u \le \tilde{u} + 0.5\,\text{cm/s}$. The power spectral density is referenced to $8.9125 \cdot 10^9$ $\mu Pa^2/Hz$. **(b)** Relation between the current speed and intensity of low-frequency noise in individual measurements is shown with black dots for the entire observation period. The intensity of pressure fluctuations is referenced to the intensity of a signal with root-mean-square pressure of 0.09441 Pa. **(c)** Same as in (b) but for the relation between current speed and centroid frequency of the pressure fluctuations spectrum. Each black dot in (b) and (c) represents a one-minute observation.

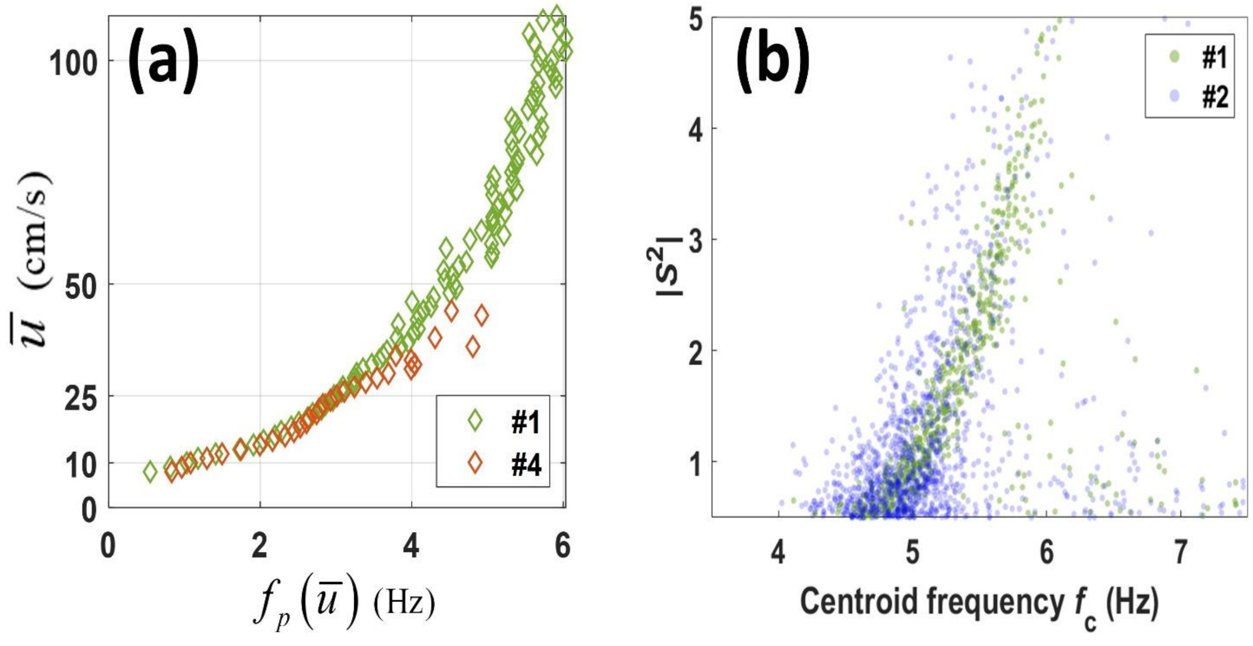

Supplementary Figure S5. Comparison of the flow noise properties at different moorings. **(a)** Relation between the peak frequency, where the averaged flow noise spectrum is maximum, and current speed at MANR#1 (green diamonds) and MANR#4 (brown diamonds). **(b)** Scatter plot of the relation between the intensity and centroid frequency of noise in the 0.5–20 Hz frequency band. Green and blue circles represent measurements at MANR#1 and MANR#2, respectively, with 1-minute averaging. The intensity of pressure fluctuations is referenced to the intensity of a signal with root-mean-square pressure of 0.09441 Pa.
